# Circular mRNA: A novel therapeutic agent

**DOI:** 10.1016/j.biotno.2023.09.001

**Published:** 2023-09-24

**Authors:** Xiaoxue Wang, Jian Dong, Yuan Lu

**Affiliations:** a Tianjin Industrial Microbiology Key Laboratory, College of Biotechnology, Tianjin University of Science and Technology, Tianjin, 300457, China; b Key Laboratory of Industrial Biocatalysis, Ministry of Education, Department of Chemical Engineering, Tsinghua University, Beijing, 100084, China

**Keywords:** Circular mRNA, *In vitro* synthesis, Enzyme ligation, Vaccine

## Abstract

Circular mRNA (circmRNA) is a covalent closed loop formed by reverse splicing of the 3′ end to the 5′ end of mRNA. Compared to traditional linear mRNAs, circmRNAs can mediate efficient, stable, and durable protein expression and are considered an alternative to linear mRNAs in terms of therapeutic reagents. With the continuous development of circmRNA research, circmRNA has also made significant progress in vaccines and cellular therapies. In this review, we present research advances in the *in vitro* synthesis of circmRNAs, focusing on the biological ligation methods of circmRNAs and current applications, with a summary of challenges regarding circmRNA design, synthesis, and applications. Based on the enhanced stability of circmRNAs, further research on circmRNAs could help expand their applications in biotherapeutics and strengthen their role in basic medical applications.

## 1 Introduction

**Fig. 1:**
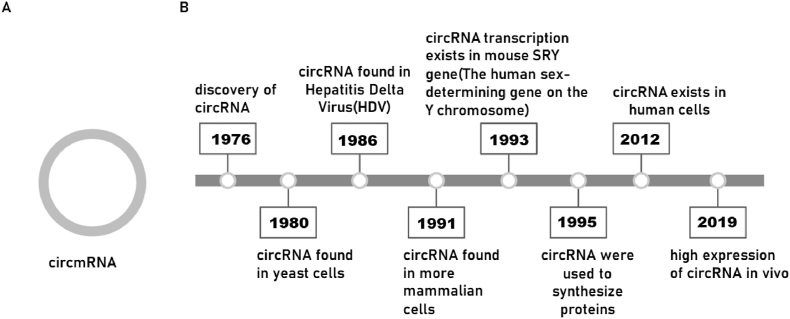
Timeline of circular RNA. (A) CircmRNA structure diagram. (B) The concept of circRNA was introduced in 1976. However, extensive research on circRNA began in 2012, when large amounts of circRNA were discovered in human cells. These studies enabled the synthesis of circRNA and expanded the application areas of circRNA.

Circluar mRNA (circmRNA) has received much attention as a novel mRNA. Compared with traditional linear mRNA, circmRNA gets a covalently closed loop by reverse splicing at the 3′ end and 5′ end, so it does not contain 5′ end cap structure and 3′ end poly (A) tail. Therefore, it does not involve the shortening of adenylate tail and 5′ end decap reaction (Fig. 1A), and the stability is much stronger than linear mRNA.^1^ The instability of linear mRNA molecules due to their short half-life and susceptibility to degradation by RNase enzymes, can be greatly alleviated.^2^

Since circmRNA lacks 5′ end-cap structure, which helps to regulate protein synthesis, most of the circmRNAs can be made to encode protein by some special structures, such as internal ribosome entry site, infinite open reading frame, etc., which opens the way for protein synthesis from circmRNAs. The design and synthesis of circmRNAs have positively affected the development of novel vaccines. Based on the current research, the understanding of circmRNAs is still at a basic stage, and a series of studies on circmRNAs can be carried out analogously to the development of circRNAs.

The first concept of circRNA was introduced by Sanger et al., in 1976, and the presence of circRNA molecules in the cytoplasm of eukaryotic cells was observed under electron microscopy.^3^ Within the next three decades, circRNA was found in many cells. circRNA was discovered in yeast cells in 1980^4^ and HDV in 1986.^5^ Starting in 1991, more circRNAs were found in mammals.^6^, ^7^, ^8^ In 1993, purely circular transcripts were also found in the Sry gene in mice, but researchers thought this was an error product of the ligation process and did not pay much attention to it. In 1995, circRNA was used to synthesize proteins. In 2012, scientists began to study loop mRNA in human cells, proving that loop mRNA is not a missplicing product but a universal feature. Efficient expression of circRNA *in vivo* was achieved in 2019 (Fig. 1B).

In this review, we present information about the *in vitro* synthesis and applications of circmRNAs, focusing on the current opportunities and challenges. Due to the unique structure and function of circmRNAs, an in-depth study of circmRNAs can help expand their applications in medicine.

## 2 CircmRNA synthesis modes

### 2.1 Preparation of linear mRNA *in vitro*

For now, *in vitro* synthesis of circmRNA is mainly done by joining the two ends of linear mRNA to obtain a covalently closed loop, which can be synthesized *in vitro* by chemical synthesis methods^9^ or enzymatic strategies.^10^ The advantage of the chemical synthesis method is that the 5 ′ end monophosphate can be introduced directly during the synthesis process for future cyclization. However, the chemical synthesis method is limited by high cost and low yield and is only applicable to RNAs of 50–70 nucleotides in length. At the same time, chemical synthesis may also have potential safety hazards and is unsuitable for widespread use. The enzymatic method is mainly used to obtain linear mRNA from linear DNA template strands by RNA polymerase, and the enzymatic method is currently the main *in vitro* synthesis method of linear mRNA in experiments.

### 2.2 Chemical ligation method

**Fig. 2:**
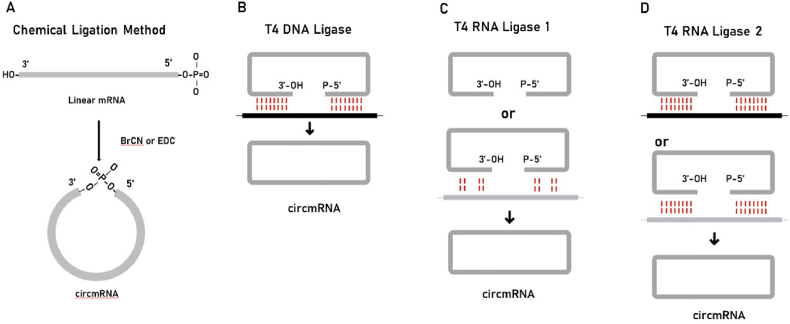
Two connection strategies. (A) Chemical ligation is achieved cyclically via cyanogen bromide (BrCN) or 1-ethyl-3- (3-dimethylaminopropyl) carbodiimide (EDC).^9^ (B) T4 DNA ligase requires ATP as a cofactor and catalyzes the formation of a phosphodiester bond between the 5′-phosphate and 3′-hydroxyl groups. The ligation will be successful only if perfect complementarity between the two strands is joined.^10^ (C) T4 RNA ligase 1 accepts DNA and RNA fragments as substrates, acts on single-stranded substrates, and has low reaction specificity.^11^ (D) T4 RNA ligase 2 ligates the RNA acceptor strand (3′-OH) to the RNA or DNA donor strand (5′-phosphate). RNA splints can be used to join the 5′-end and 3′-end together.^12^

Chemical ligation of circmRNAs is achieved by inducing 5′ terminal phosphates on linear RNAs to link the 3′ terminal hydroxyl groups. The catalysts such as ethyl-3-(3-dimethylmethylpropyl) carbodiimide (EDC) are used for phosphodiester bond formation (Fig. 2A). However, there is a significant problem with the chemical ligation method. The product obtained may not be the target product we want, and the phosphate migration may form a 2′-5′ phosphate bond instead of the natural 3′-5′ phosphodiester bond,^9^ leading to a decrease in yield. The most significant advantage of the chemical ligation method is that it can easily combine multiple types of modified residues with higher purity. Still, it is not the best choice for *in vitro* synthesis of circmRNA.

### 2.3 Biosynthesis method

#### 2.3.1 T4 ligase

Enzyme ligation is used to achieve *in vitro* synthesis of circmRNA through catalysis by several enzymes from T4 phage. T4 DNA ligase (T4 Dnl),^10^ T4 RNA ligase 1 (T4 Rnl 1),^11^ and T4 RNA ligase 2 (T4 Rnl 2) are commonly used.^12^^,^^13^ T4 DNA ligase catalyzes the formation of phosphodiester bonds between 5' -phosphate and 3′-hydroxyl groups via ATP as a cofactor. T4 RNA ligase 1 accepts DNA and RNA fragments as substrates and acts on single-stranded substrates with low reaction specificity. T4 RNA ligase 2 connects the RNA acceptor strand (3′-OH) to the RNA or DNA donor strand (5′-phosphate) by transferring 3 nucleotides, allowing the 3' –OH of the acceptor substrate and the 5′-phosphate group of the donor substrate to join^14^. T4 DNA ligase 1 and T4 RNA ligase 1 are more suitable for ligation of mRNAs without complex secondary structures, and T4 RNA ligase 2 is more suitable for linear mRNA precursors with ligation sites at both ends. Therefore, the appropriate enzyme linkage method should be selected according to the different linear mRNA precursors.

T4 DNA ligase forms phosphodiester bonds by linking the 5′ end phosphate to the 3′ end hydroxyl group in double-stranded regions of DNA or RNA or even in DNA-RNA hybrid structures (Fig. 2B). To improve the ligation accuracy, a complementary DNA template is required for RNA ligation.^15^^,^^16^ This hybridized structure can be recognized by T4 Dnl to obtain cyclized mRNA, and the reacted DNA template can be degraded with DNase and then the circmRNA can be separated from the linear precursor by electrophoresis. In addition, the T4 Dnl method is inefficient and often requires a large amount of enzymes for RNA ligation, especially when compared to specialized RNA ligases.^17^ Due to these characteristics, only a few experiments will choose this method for RNA ligation.

T4 RNA ligase 1 is a more widely used RNA ligase. T4 Rnl 1 catalyzes the nucleophilic attack of the 3′-OH terminus on the activated 5′-terminus to form a covalent 5′,3′-phosphodiester bond to form a circmRNA^17^ (Fig. 2B). However, the efficiency of the reaction is largely dependent on the structure of the linear precursor. In addition, the 3′-terminus and 5′-terminus have an inconsistent preference for different nucleotides. 3′-terminus nucleotide preference is in the order of A > G ≥ C > U, and 5′-terminus nucleotide preference is in the order of C > U > A > G.^4^^,^^15^^,^^18^, ^19^, ^20^ The circmRNA obtained by ligation requires only 6–8 nucleotides, which can achieve efficient single-stranded mRNA linkage. The corresponding ligation efficiency decreases when the RNA molecule is larger. As with T4 DNA ligase, intermolecular end-joining is a side reaction that cannot be completely eliminated and inhibits the formation of circmRNAs.

T4 RNA ligase 2 can also be used for the cyclization of mRNA by catalyzing the nucleophilic attack of the hydroxyl terminus of the 3′ end of the acceptor strand on the activated 5′ end of the donor strand, forming a covalent phosphodiester bond^12^(Fig. 2D). T4 Rnl 2 is more active and efficient in joining gaps in double-stranded RNA. When used with a 20 nucleotide DNA template, the efficiency of T4 Rnl 2 and T4 Dnl is comparable. However, in the absence of templates, the efficiency of RNA ligase remains constant, and that of DNA ligase decreases.^21^ T4 Rnl 2 can be used for the ligation of single-stranded RNA by setting up RNA splints, the stability of which is disturbed when ligating short RNA precursors. Similarly, T4 Rnl 2 suffers from low efficiency for the ligation of large RNAs and side reactions.

In conclusion, the selection of different T4 ligases needs to be based on the secondary structure of the linear RNA precursor. At the same time, there are also problems that the connection of macromolecular RNA cannot be realized. The side reactions of terminal connections between molecules cannot be avoided entirely, and these problems still need to be solved.

#### 2.3.2 Ribozyme method

The most popular of the ribozyme methods is permuted introns and exons (PIE). The PIE method can be used for *in vivo* and *in vitro* mRNA circularization,^22^ which improves the expression of circmRNA.

**Fig. 3:**
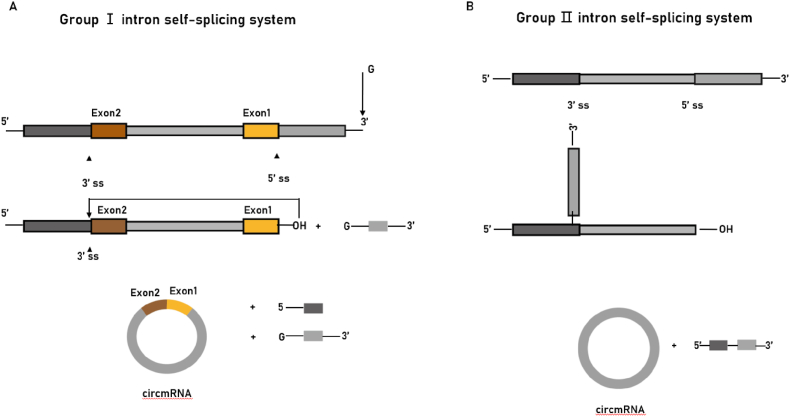
Strategies of ribozyme methods. (A) Group I intron self-splicing system requires only the addition of GTP and Mg^2+^ as cofactors and shows great potential for protein synthesis. This method realized mRNA ligation through a regular group I intron self-splicing reaction, including two transesterifications at defined splice sites. The final circmRNA will contain exogenous exon sequences. (B) Group II intron self-splicing system involves the joining of the 5′ splice site at the end of an exon to the 3′ splice site at the beginning of the same exon. All exon sequences are dispensable for group II intron-catalyzed inverse splicing. This method can enable more accurate linear RNA precursor ligation.

The modified group I intron self-splicing PIE method is the most common ribozyme method.^23^ Group I intron self-splicing method divides the mRNA intron and the auxiliary exon fragment into two parts, where the 5′ end sequence is transferred to the tail of the target sequence and the 3′ segment sequence is inserted into the front of the target sequence (Fig. 3A).^23^^,^^24^ The assistance of GTP and Mg^2+^ leads to the autocyclization of sequences other than introns. Compared with chemical ligation methods as well as enzymatic ligation methods, this method can be used for the cyclization of larger linear mRNA precursors with efficient and accurate ligation. A recent study achieved precise ligation by designing specific transcriptional templates, meaning that synthetic circmRNAs of almost all sequences can be efficiently synthesized without exogenous exon sequences.^25^ Based on these advantages, the group I intron self-splicing method is widely used, but the system still has unavoidable drawbacks. The introduction of exogenous sequences leads to the fact that the sequence of the resulting circmRNA will differ from the original precursor linear mRNA sequence.

The PIE method based on group II intron self-splicing can also be used as a nuclease approach to circmRNA synthesis.^26^ Through the inversion of the Exon1 and Exon2 sequences in the six structural regions of the type II intron, a self-splicing active ribozyme can be formed to prepare circmRNA *in vitro* (Fig. 3B). Compared with the type I intron PIE system, the biggest difference of the type II intron is that it can synthesize circmRNA without the introduction of splicing scars. There is no residue of foreign sequences, which is suitable for producing circmRNA with precise sequences.^27^^,^^28^ However, this method forms a 2′,5′-phosphodiester bond at the linking site instead of a natural 3′, 5′-phosphodiester bond. The mechanism of mRNA is unclear and still controversial.^4^^,^^29^

**Fig. 4:**
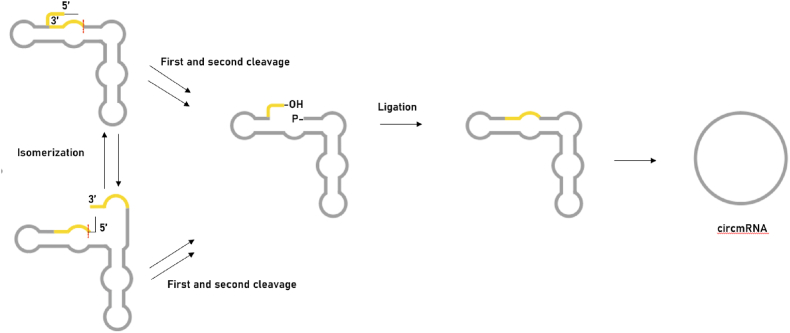
The strategy of the Hairpin ribozyme method. Hairpin ribozyme method can produce circmRNA through the rolling circle and self-splicing reactions. The linear mRNA precursor with HPRs can fold into two alternative cleavage-active conformations to remove the 3′-end and the 5′-end. As a result, the intermediate can contain a 5′-OH and a 2′, 3′-cyclic phosphate to produce circmRNA.

The intrinsic cleavage and ligation activities of hairpin nucleases (HPRs)^13^^,^^30^ also inspire the targeted synthesis of circmRNAs *in vitro* (Fig. 4). CircmRNAs are mainly generated by rolling loop reactions and self-shearing reactions. Stable structures of HPRs facilitate linkage and easy fragment retention binding, and less stable HPRs facilitate cleavage and are more conducive to the dissociation of cleaved fragments. HPRs structures can be embedded in variable-length mRNAs, thus paving the way for the preparation of circmRNAs.^31^ The linear mRNA precursor with HPRs will fold into two selective active conformations, severing the 3′ end and the 5′ end. The intermediate thus contains a 5′-OH and a 2′,3′-circular phosphate to produce the target circmRNA. In contrast to the previously mentioned methods for circ RNA synthesis, this strategy is suitable for creating circmRNAs with uniform sequences at the junction.^32^ However, this system is not widely used in this field, probably due to the following drawbacks: ⅰ) the HPRs sequence remains embedded in the final circmRNA, which may have negative effects; ii) the resulting circmRNA itself retains active nuclease activity, leading to oscillation between linear and circular and is not stable; (iii) upon introduction into the cell, the circular containing these sequences mRNAs may cleave other RNAs in trans, leading to off-target effects.

Of all nuclease-related *in vitro* synthesis methods, the PIE method using group I introns shows the greatest potential for circmRNA production. In contrast to other ligation methods, this method does not require splinting, does not take into account the secondary structure of the linear precursor, and is effective in enhancing circmRNA expression. The PIE method of group I intron can achieve more durable protein expression *in vitro*, which is very suitable for *in vivo* application as a vaccine or therapeutic drug, but it is easy to generate by-products, such as exon fragments of introns produced by splicing reactions, making it challenging to obtain high-purity circmRNA. The PIE method of group II introns can achieve more precise ligation of linear precursors, but the *in vitro* mechanism is still unclear. Hairpin ribozyme methods are susceptible to foreign sequences. At present, the PIE method has been widely used in research and industrial production (Table 1).

**Table 1:** Comparison of different *in vitro* mRNA synthesis methods.

Cyclization method	Advantages	Disadvantages
Chemical linkage	Multiple chemical reactions No intron scar Suitable for short fragments (below 100 nt)	Cause the formation of non-3′-5′ phosphodiester linkages Toxic Need to use DNA splint The steps are complicated
T4 DNA ligase	The sequence is relatively long Can produce precise sequences With or without splints	Need to optimize splint sequence Low turnover efficiency and large usage
T4 RNA ligase 1		Circularization efficiency depends on linear RNA secondary structure Nucleotide preference for terminal nucleotides
T4 RNA ligase 2		Circularization efficiency depends on the secondary structure of linear mRNA
PIE system - type Ⅰ intron	Wide range of application length (100 nt∼5 kb)	Retention of helper exons in the PIE structure
PIE system - type Ⅱ intron	Produce a precise sequence	The mechanism is unclear
Hairpin ribozymes	Suitable for short fragments (50∼150 nt)	Contains ribozyme sequences Has potential ribozyme activity

## 3 Applications of CircmRNA

The fact that circmRNAs outperform linear mRNAs in terms of conformation, stability, and immunogenicity has prompted attempts to develop circmRNA-based technologies. CircmRNAs can be the material of choice for vaccines because they are less susceptible to degradation, more stable than linear mRNAs, less toxic, etc.^33^^,^^34^ The development of nucleic acid vaccines has attracted widespread attention, and research on the application of circmRNA is now increasingly urgent.

### 3.1 CircmRNA in infectious disease vaccines

Researchers at Mahidol University in Thailand developed a circmRNA expressing SARS-CoV-2 spike as a vaccine protein capable of neutralizing broad SARS-CoV-2 variants.^35^ It has been reported that a promising candidate for the next-generation SARS-CoV-2 vaccine is the spike protein with six rationally substituted amino acids to reflect emerging variants. This finding highlights the importance of ongoing research and development efforts to address the evolving nature of the virus and protect public health.

To enhance the broad-spectrum protection of the vaccine, Wei et al. attempted to synthesize circmRNA *in vitro* using T4 RNA ligase by fusing the trimeric motif of phage T4 fibronectin to the C-terminus of the RBD to produce a high concentration and abundance of the antigen.^36^ This research showed that the circmRNA vaccine induced a sustained humoral immune response, producing high levels of neutralizing antibodies, providing effective protection to the organism, and making it an effective option against novel coronavirus variants. It is also suggested that the *in vivo* immunogenicity and biosafety of *in vitro* prepared circmRNAs still need further validation.

### 3.2 CircmRNA in cancer

With the development of bio-sequencing technology, more and more circmRNAs are specifically expressed in cancer, and researchers have discovered that circmRNAs translate proteins and are involved in regulating a variety of cancer diseases and play essential functions in the development and progression of cancer diseases. The discovery of these theoretical studies will potentially provide strong support for cancer therapy.

#### 3.2.1 Glioma tumorigenesis

Glioma tumorigenesis is the most common and most malignant primary tumor in the brain. The researcher from Sun Yat-sen University demonstrated for the first time that circmRNA-translated protein can regulate the occurrence and development of glioma.^37^ circFBXW7 suppresses the cell cycle of malignant glioma by translating 21 kD protein and regulating the stability of the proto-oncogene c-Myc protein. In addition, circPINT can be translated into an 87 amino acid polypeptide, which can act as a glioma suppressor and has potential value in clinical studies of glioma disease.^38^ Researchers also found that circAKT3 can keep glioma cell proliferation, apoptosis, and tumor formation *in vitro* and *in vivo*, confirming that circAKT3 can be used as a prognostic marker for glioma.^39^ Zhang et al. reported that circSHPRH-translated protein expression was downregulated in glioma patients compared to normal subjects, demonstrating its correlation with the degree of malignancy of glioma.^40^

#### 3.2.2 Colon cancer

Colon cancer is the fourth most life-threatening disease in humans worldwide. A 73-amino acid peptide encoded by circPPP_1_R_12_A, is associated with colon cancer and has potential roles in colon cancer.^41^ Patients with higher levels of circPPP_1_R_12_A had significantly shorter overall survival. CircPPP_1_R_12_A played a critical role in the proliferation, migration, and invasion of colon cancer cells. This study has identified that circPPP_1_R_12_A-73aa stimulated the tumor pathogenesis and metastasis of colon cancer via activating Hippo-YAP signaling pathway. The findings of this research suggest that this discovery could potentially lead to the development of new and effective treatment options for individuals with colon cancer.

#### 3.2.3 Gastric cancer

Gastric cancer is one of the most common malignancies worldwide. In the past decades, the incidence and mortality of gastric cancer have remained high despite tremendous efforts to improve the diagnosis and treatment of gastric cancer. Recent studies have identified a circMAPK1 that is lowly expressed in gastric cancer, and this circmRNA-translated protein affects cancer development by competing with MAPK1 for binding sites, thereby inhibiting MAPK1 expression and phosphorylation of extremely downstream genes.^42^ In addition, circDIDO1-translated protein plays a vital role in the development and progression of gastric cancer, which can interact with PARP1 and inhibit its activity. This study suggests that circDIDO1 is expected to be a potential prognostic biomarker and therapeutic target.^43^

#### 3.2.4 Cervical cancer

Cervical cancer is the second most common cancer in women worldwide, and infection with certain high-risk HPV (human papillomavirus) types is the greatest risk factor for cervical cancer. Studies have shown that HPV can produce a variety of circmRNAs, including circE7. This circmRNA drives translation through m6A modifying action to produce the E7 oncoprotein. It was found that circE7 inhibits cancer cell growth by reducing the level of E7 oncoprotein in cervical cancer cells. Whether circE7-translated protein serves as a sensitive marker for changing to HPV population and circE7 abundance has prognostic significance in cervical cancer, still needs to be further investigated.^44^

#### 3.2.5 Breast cancer

Breast cancer is the most common cancer in women. Recent studies have found that circ-HER2 and its translational proteins HER2-103 are highly expressed in clinical samples.^45^ It is important to note that triple negative breast cancer (TNBC) patients who test positive for HER2-103 tend to have a worse overall prognosis than those who test negative. This is due to the critical role that HER2-103 plays in TNBC tumorigenicity. The study emphasized the potential clinical benefits of Pertuzumab in treating TNBC patients who express circ-HER2/HER2-103.

#### 3.2.6 Multiple myeloma

Multiple myeloma (MM) is a molecularly and cytogenetically heterogeneous hematologic malignancy of bone marrow origin, characterized by chromosomal instability. The expression of BUB1B translated by circBUB1B increased strikingly in MM patients, facilitated cellular proliferation, and induced drug resistance *in vitro* and *in vivo*, which was closely correlated with poor outcomes.^46^ *In vitro* and *in vivo* studies have shown that circBUB1B significantly inhibits the malignancy of multiple myeloma. It appears that promising therapeutic targets for MM include BUB1B or circBUB1B.

Many circmRNA companies have been founded and developed, including Orna Therapeutics, Laronde, circmRNA Biology, Roundin Biology, Gisai Biology, and CoreMed. However, no circmRNA-based industrial-grade cancer vaccine has been created yet, so the applicability of circmRNA vaccines in tumor immunotherapy needs to be further explored.

## 4 Prospects and challenges

Despite the increasing interest in circmRNAs, many challenges and opportunities remain. Our understanding of circmRNA is still the tip of the iceberg, and elucidating its biological function and mechanism is still far away.

For circmRNA, we still face significant challenges with the current technology. The first is the challenge of synthesis (Table 2). The first point is that *in vitro* transcribed circmRNA has some problems that lead to low synthesis efficiency. Although the PIE method can achieve the correct ligation of linear mRNA precursors, the precursor structure still significantly impacts the ligation accuracy. Trying to embed non-natural nucleotides into linear mRNA precursors to change the secondary structure may help improve ligation efficiency. The second point is that enzymes and other reagents are expensive and have low cyclization efficiency. Existing phage T4 ligase has the natural ligating ability, but there is still much room for improvement. The cyclization efficiency can be improved by effective mutation and rational design. By controlling the concentration of linear mRNA precursors, the occurrence of side reactions might be reduced. The third point is that the purity of the obtained circmRNA needs to be improved. Many different RNA species were isolated from the circmRNA obtained by the PIE method, so the purification method needs further improvement.

**Table 2:** Problems faced by synthetic circmRNA.

Challenges	Possible solutions
Low cyclization efficiency for large RNA molecules	Well-designed structure
Intermolecular side reactions, especially the end-joining part	Optimizing the cyclization reaction conditions and controlling the linear RNA precursor concentration
Generation of modified circmRNA	Incorporating chemically modified groups or unnatural nucleotides during the ligation reaction
The yield of circmRNA	Optimizing current reaction components and conditions
Cost issues	Synthesis of nucleotides using cells or direct synthesis of linear mRNA precursors
Untapped potential	Transferring scientific research achievements to commercial application

Although circmRNAs have proven potential as vaccines, they have not been fully exploited. Due to their unique structures, some specific proteins can be synthesized. In addition, circmRNA can also be used as a biomarker for certain diseases. Since some functions and mechanisms of circmRNAs are not well understood, further efforts are needed. Regardless of the specific role of circmRNA, it still holds great potential. With the continuous efforts of researchers, mRNA will play a more important role and become an important part of human health in the future.

## Declaration of competing interest

The authors declare that they have no known competing financial interests or personal relationships that could have appeared to influence the work reported in this paper. Yuan Lu is the Editor-in-Chief for *Biotechnology Notes* and was not involved in the editorial review or the decision to publish this article.
